# Evolution of the Complex *Growth Hormone* Gene Cluster in Macaques

**DOI:** 10.1210/endocr/bqaf056

**Published:** 2025-03-19

**Authors:** Michael Wallis

**Affiliations:** Department of Biochemistry and Biomedicine, School of Life Sciences, University of Sussex, Brighton BN1 9QG, UK

**Keywords:** growth hormone locus, gene cluster, macaque, placental lactogen, molecular evolution, gene duplication

## Abstract

In higher primates, unlike other mammals, the *GH* gene locus is complex, comprising several *GH*-like genes, resulting from gene duplication and divergent evolution, expressed in pituitary and placenta. There are 5 genes in this *GH* gene cluster in human and 5 to 7 in apes and most Old-World monkeys, but in macaques the cluster has expanded further. Here the nature and evolution of the *GH* locus in this important primate genus is explored. Analysis of genomic data for *Macaca fascicularis* (crab-eating macaque) revealed that the *GH* gene cluster in this species is variable, with at least 5 different haplotypes, comprising 11 to 14 *GH*-like genes. Gene-number heterozygosity was also detected in *Macaca mulatta* (rhesus macaque) with 9 to 13 genes. Analysis of genomic data for other macaque species revealed *GH* gene clusters containing 8 to 14 *GH*-like genes, but gene-number heterozygosity was not detected. Expression of *GH*-like genes in pituitary and placenta was examined for *Macaca fascicularis.* This analysis has established that the complexity of the *GH* gene cluster increased during the evolution of macaques, by gene duplication and divergent evolution, and that these processes continue within at least 2 extant species. Analysis of rate of sequence change, and distribution of substitutions within the 3D structure, shows that for at least 1 *GH*-like gene (*GH2*), the changes reflect positive selection, implying adaptive biological change. Whether this involves changes in physiological (endocrine) function or response to viral or other pathogenic challenge is not yet clear.

In most mammals, the *GH* locus comprises a single gene for pituitary GH, flanked by unrelated 5′ (normally *CD79B*) and 3′ (normally *TCAM1P* and *SMARCD2*) genes (1). In higher primates, however, the locus has expanded to include a cluster of closely related genes, still flanked by 5′ *CD79B* and 3′ *TCAM1P* (2-6). This gene cluster has apparently been produced during the course of evolution by repeated gene duplications and arose independently in Old World Monkeys (OWM)/apes and New-World Monkeys (NWM) (5, 7, 8). In human, the cluster comprises 5 genes, with that encoding pituitary GH at the 5′ end, followed by 4 genes expressed in placenta, 2 encoding placental lactogen [PL; also known as choriosomatomammotropin hormone (CSH)], 1 encoding GH variant and 1 a PL-related gene, probably effectively a pseudogene (2).

The biological role of these placental genes and their protein products is unclear (9, 10). They are expressed by the fetal side of the placenta. PL levels in human maternal blood rise to very high levels during pregnancy (1-2 µg/mL, approximately 100-fold greater than circulating levels of GH in nonpregnant individuals) and fall to undetectable within 1 to 2 days of birth. A role in the regulation of growth and development of the mammary gland is possible, though the hormones, including prolactin, that carry out this function in nonprimates are also present in human. A role in regulating the balance of nutrition between mother and fetus, which may involve maternal-fetal conflict (11), is also possible. A complicating observation is that in human a few cases of deletion of placental lactogen genes have been observed, with complete lack of circulating PL, but no apparent deleterious effects on either mother or fetus/offspring (12). It has also been suggested that PLs may play a role in protecting the fetus against pathogens (13). The human placental GH variant circulates at moderate (∼10-20 ng/mL) levels in the maternal blood and appears to replace pituitary GH, which falls to undetectable levels during pregnancy (14, 15).

In other primates, the number of genes in the *GH* cluster varies. In apes there are 5 in chimpanzee (3), 6 in gorilla, 4 in orangutan (4), and 7 in gibbon (16, 17) (divided in the last case between 2 chromosomes); in each case, the *GH* gene is at the 5′-end of the cluster. In NWM, there are 8 *GH*-like genes in marmoset (5) and a large number (at least 20) in *Cebus* spp (7). In OWM, the number of genes is also quite variable. A cluster of 6 or 7 *GH*-related genes was originally reported in rhesus macaque (18), but more-recently genome sequences reported in the Ensembl and NCBI genomic databases suggest up to 13 genes in the cluster. Genome sequences for some other macaque species also suggest 10 or more genes in the cluster, but in other OWM the number is only 5 to 7, including the species most closely related to macaques, such as baboon and mandrill (6, 19). In both OWM and NWM, the gene at the 5′ end of the cluster encodes pituitary GH, and placental expression of the other genes has been demonstrated in some species. In OWM, several of the placentally expressed genes are equivalent to the PLs seen in human and apes, indicating that they arose as the result of an initial gene duplication preceding diversion of OWM and apes. However, this is not the case for NWM, where an independent initial gene duplication appears to have given rise to the *GH* gene cluster (5, 7, 8).

The *GH* gene cluster in macaques is clearly large and complex, but its detailed organization remains uncertain. Given the importance of this group as a widely studied primate model, and the fact that human placental lactogens only have homologs in higher primates, clarification of this situation is important. The object of the present study was to analyze available genomic data to obtain a clearer picture of the *GH* gene cluster in macaques. It was established that in crab-eating macaque, *Macaca fascicularis* (*M. fascicularis*), the cluster exists as a number of different haplotypes, containing 11, 12, 13, or 14 *GH*-related genes. Rhesus macaque [*Macaca mulatta* (*M. mulatta*)] also shows gene-number heterozygosity, with haplotypes containing 9, 12, or 13 genes. In other *Macaca* species, clusters of 8 to 14 *GH*-like genes were detected but without clear evidence of gene-number heterozygosity. The nature of the encoded proteins and their expression in placenta and pituitary were explored. It has been shown previously that rapid evolution, driven by positive selection, has occurred widely in higher primate *GH*-like genes, and it is clear that this has continued within the *Macaca* genus, with further gene duplication and diversification.

## Methods

### Data Sources

Macaque genomic assemblies were obtained from the NCBI (https://www.ncbi.nlm.nih.gov/) and Ensembl (https://www.ensembl.org/) websites. In most cases, the region including the macaque *GH* gene cluster was too incomplete, inconsistent, and/or inaccurate to provide a satisfactory basis for the study described here. The nature of these gene clusters was therefore investigated by analysis of original sequences obtained from the NCBI Sequence Read Archive (SRA) using Blast searches (20). Long reads (Oxford Nanopore or PacBio) that cover a substantial part of the *GH* cluster were particularly useful where available. Details of sources used are given in Supplementary Table S1 (21).

Expression was examined using transcriptomic data for pituitary or placenta obtained through the SRA. Sources used are given in Supplementary Table S1 (21). Transcriptomes were subjected to Blast analysis using appropriate whole coding sequences (CDS) or shorter (80 nt) sequences that allowed discrimination between the various GH-like transcripts. In many cases, the high expression rate made it necessary to restrict the number of reads searched, which was done by downloading and analyzing a limited number of reads (usually 100 000) from a transcriptome.

Protein sequences were derived by conceptual translation of corresponding coding sequence. They are represented using the standard 1-letter code for amino acid residues.

### Evolutionary Analysis

Phylogenetic trees were constructed using PAUP* (22) and modified on the basis of previous studies on *Macaca* phylogeny (23, 24).

Analysis of adaptive evolution using dN/dS ratios was carried out using the branch model of the CODEML program in the PAML package (25, 26). The significance of accelerated evolution on a specific branch of the phylogeny was determined using the likelihood ratio test, comparing the likelihood ratio test statistic (2xΔlnL) with the chi-square distribution.

### Molecular Modeling

A molecular model of *M. fascicularis* GH2a was produced using AlphaFold 3 (27) and manipulated in PyMOL (The PyMOL Molecular Graphics System, Version 2.5.0 Schrödinger, LLC). Potential receptor-binding sites were determined using the 3D structure of human GH bound to dimeric receptor given in PDB file 3hhr (28). Amino acid residues changing on specific branches of the evolutionary tree were determined using the APOLIST option in PAUP* and mapped onto the molecular model using PyMOL.

### Nomenclature

The GH-like proteins expressed in the placenta of higher primates have been referred to variously as GH variant (GH-V or GH2; most closely similar to GH) and PL or CSH. Golos et al (29) reported the cloning of 4 GH-like cDNAs from placenta of rhesus macaque; these were designated mGH-V, mCS1, mCS2, and mCS3, with mCS1 and mCS2 being very similar. González Álvarez et al (18), investigating the genomic organization of the *GH* gene cluster in this species, identified 6 genes, pituitary *GH*, *GH2* (corresponding to mGH-V of Golos), *CSH1* (corresponding to mCS1/2), *CSH2* (corresponding to mCS3), *CSH3* (not previously reported, apparently a pseudogene), and *CSH4* (not previously reported). It is now recognized that the gene cluster contains more than 6 genes due to recent duplications of *CSH2*, *GH2,* and *CSH4*. The nomenclature used in this paper is based on that of González Álvarez et al (18), with the multiple copies of the last 3 genes distinguished by letters (eg, *GH2a*, *GH2b*, *GH2c*). *GH*-like genes were designated as *GH2* or *CSH1-4* on the basis of similarity to the forms originally identified by Golos et al (29).

## Results

### The *GH* Gene Cluster in *M. fascicularis*

Genomic assemblies available for *M. fascicularis* show a large *GH* gene cluster on chromosome 16, but the size of the cluster seems uncertain. Thus, it comprises 11 genes in Ensembl assembly 6.0 but 13 genes in NCBI RefSeq GCF_037993035.1 (assembly T2T-MFA8v1.0). Other NCBI assemblies show from 5 to 18 genes in the cluster. This *GH* locus is clearly complex but in need of clarification. Studies on original sequence data obtained from the SRA were therefore carried out to address this need.

**Figure 1.**
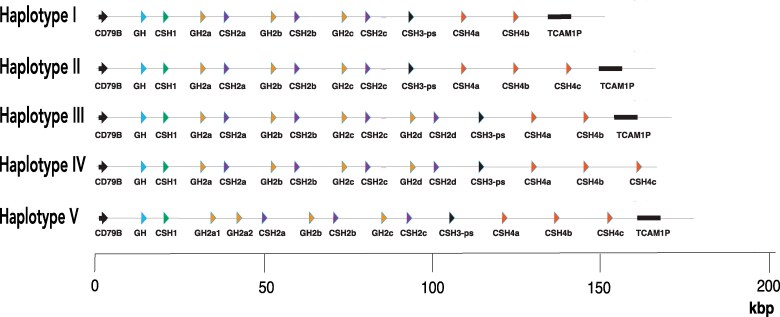
Five haplotypes for the *GH* gene cluster in *Macaca fascicularis.* For each haplotype, the 11–14 *GH*-like genes are represented as triangles, distinguished by color and labeling. The gene for pituitary GH is at the 5′ end of the cluster (left-hand side) in every case. Flanking genes are also included: *CD79B* (5′) and *TCAM1P* (pseudogene) (3′). Haplotypes I to IV were characterized using data from the Sequence Read Archive project SRP384596. Haplotype V is that in the NCBI reference genome T2T-MFA8v1.0.

SRA project SRP384596 involves the whole-genome sequencing of 13 individual Mauritian-origin *M. fascicularis* and includes some very long sequence reads (>100 kb; Oxford Nanopore), potentially including the entire *GH* gene cluster in a single read. Several such reads were identified that included gene sequences flanking both the 5′ and 3′ ends of the *GH* cluster. The sequences included are rather inaccurate, due particularly to the presence of numerous small insertions or deletions (indels) but can define accurately the number of genes in the cluster and their overall organization. Examination of such long reads from several individual animals revealed that there were at least 4 haplotypes for the *GH* gene cluster, including, respectively, 11, 12, 13, and at least 14 *GH*-like genes, The organization of these is shown in Fig. 1. If the 11-gene form is taken as the basic structure, the other haplotypes have been produced by duplication of the last gene in the cluster (*CSH4*), duplication of a pair of genes (*GH2-CSH2*) within the cluster, or both of these. The numbers and nature of the full-length (including defined flanking regions for each end of the cluster) or almost full-length sequences are given in Table 1, which suggests that individual animals are homozygous or heterozygous. Given the small numbers, some heterozygotes may have been missed, but this does not affect the overall conclusion regarding the presence of multiple gene-number haplotypes. Examination of more accurate sequences (see next paragraph) supported the results.

**Table 1.** Occurrence of *GH* gene cluster haplotypes in individual crab-eating macaques

Individual	ONT run		Haplotypes present*^a^*	Biopac run
cy0333	SRX15982296	SRR19939831	Type I (1), Type II (1)	SRX15982297
cy0161	SRX22333527	SRR26633167	Type II (4)	SRX22333528
cy0390	SRX22333529	SRR26633165	Type I (2), Type III or IV (1)	SRX22333530
cy0424	SRX22333531	SRR26633163	Type I (2), Type II (2)	SRX22333532
cy0558	SRX22333533	SRR26633161	Type I (1), Type II (1)	SRX22333554
cy0568	SRX22333535	SRR26633159	—	SRX22333536
cy0692	SRX22333537	SRR26633157	Type I (2), Type III or IV (1)	SRX22333538
cy0322	SRX22333539	SRR26633155	Type III (1)	SRX22333544
cy0695	SRX22333540	SRR26633154	Type II (3), Type III or IV (1)	SRX22333541
cy0973	SRX22333542	SRR26633152	—	SRX22333543
cy0325	SRX22333545	SRR26633149	Type II (3)	SRX22333546
cy0330	SRX22333547	SRR26633147	Type I (1)	SRX22333548
cy0355	SRX22333549	SRR26633145	Type IV (1)	SRX22333550

^*a*^Numbers in parentheses indicate numbers of reads containing full or almost full sequences for the haplotype indicated.

For each of the animals listed in Table 1, sequence data were available from both very long reads (Oxford Nanopore) and more accurate long reads (PacBio). For animals that were homozygous, it was feasible to use the latter data to assemble an accurate sequence on the basis of the overall organization provided by the former. This was done for animal cy0325, for which analysis of Oxford Nanopore and PacBio data gave no indication of heterozygosity. The complete sequence derived for the 12-gene form of the *GH* gene cluster (haplotype II) is available in the Third Party Annotation Section of the DDBJ/ENA/GenBank databases under accession number TPA: BK069932. Analysis of protein sequences derived from the 12 genes in the cluster is given later. The cluster extends over about 150 000 base pairs, with the gene encoding GH at the 5′ end. Sequence similarity between the gene pairs *GH2a-CSH2a, GH2b-CSH2b,* and *GH2c-CSH2c* is very high (>99% identity) as is similarity between *CSH4a, CSH4b,* and *CSH4c* and surrounding sequences. Like the human *GH* gene cluster, that of *M. fascicularis* includes many copies of the *Alu* SINE repetitive element, most notably a very recent insertion into intron 1 of *CSH4b*, apparently reflecting the active *Alu* that has been identified in this species (23).

The occurrence of several haplotypes could explain at least some of the differences between the various Ensembl and NCBI genome assemblies, and these were reexamined in this light. The organization of the *GH* locus in the Ensembl assembly (release 6.0) suggests that the 11-gene *GH* cluster here corresponds to haplotype I, although the presence of several indels in the coding sequences of the last 2 genes suggests sequencing errors, confirming that they did not provide an adequate basis for the analysis carried out in the present study. The NCBI RefSeq assembly includes a 13-gene *GH* cluster, but the organization of this is different from that of haplotype III, corresponding to that of haplotype II with an additional *GH2* gene. This RefSeq assembly (assembly T2T-MFA8v1.0) is based on a haploid cell line (MFA582-1) and on very long sequence reads (Oxford Nanopore and PacBio; SRA project SRP475252), some containing the entire *GH* gene cluster. Analysis of these confirmed the organization of the *GH* cluster shown in the genome assembly, which is shown in Fig. 1, as haplotype V. Thus 5 different haplotypes for the *M. fascicularis GH* gene cluster have been identified.

### Proteins Encoded by the *GH*-like Genes of *M. fascicularis*

**Figure 2.**
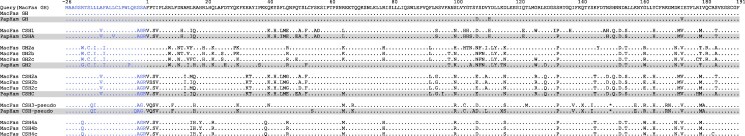
Alignment of GH-like protein sequences from *Macaca fascicularis* and *Papio hamadryas* (baboon; shaded grey). The sequence of *Macaca fascicularis* GH was used as Query; other sequences are compared with this, shown with dots for identities. Signal peptides are numbered −26 to −1 (shown in blue). MacFas *CSH3* and PapHam *CSH-pseudo* are probably pseudogenes, unlikely to be translated; their sequences have been “forced into frame” by introducing single nucleotide insertions at “X” and ignoring stop codons (*).

Sequences for the GH-like proteins of *M. fascicularis* haplotype II were obtained by conceptual translation; an alignment is shown in Fig. 2, with GH as the reference (Query) sequence. In accordance with the similarity between gene sequences noted earlier, the proteins encoded by CSH2a/CSH2b/CSH2c and CSH4a/CSH4b/CSH4c are very similar, with the CSH2 group showing differences at 3 sites and the CSH4 group differences at only 1 site. The GH2a/GH2b/GH2c group is more variable, with differences at 16 sites. Comparison with baboon (19) shows that CSH1 is equivalent to Pha CSHA, GH2a/GH2b/GH2c to Pha GH2, and CSH2a/CSH2b/CSH2c to Pha CSHC. *CSH3*-pseudo includes a single base deletion in exon 5 and consequent frame shift mutation, as well as a stop codon, preventing translation to give a normal CSH protein. It appears to correspond to the pseudogene in baboon, which has equivalent mutations and stop codon but also an additional single base deletion. CSH4a/CSH4b/CSH4c has no equivalent in baboon but corresponds to CSH4 reported previously for *M. mulatta* (18). Protein sequences corresponding to CSH1, GH2, CSH2, and CSH3-pseudo were reported by González Álvarez et al (18) for *M. mulatta*, and equivalent sequences derived by computer prediction are included in the NCBI database but do not take full account of the additional duplicated sequences now recognized. The situation in this species is considered further later on.

### Expression of the *GH*-like Genes in *M. fascicularis*

Expression of the *GH*-like genes was examined by analyzing transcriptome databases (containing original sequence data) available for pituitary and placenta as described in the Methods (Data Sources) section. Based on analysis of SRA project SRP410543 (30), expression of GH in pituitary was high (∼1% of all transcripts), as expected, with no difference between male (1.05 ± 0.09% of all transcripts, n = 3) and female (0.97 ± 0.19% of all transcripts, n = 3) animals. Very low expression (<0.0001% of all transcripts) of *CSH1* was observed in pituitary and no expression of the other *GH*-like genes. Whether this very low expression of *CSH1* in the pituitary is of physiological significance is unclear, it may reflect a trivial “spill-over effect” from the highly expressed *GH* gene immediately upstream.

In placental tissue, expression of *GH*-like (but not *GH*) genes increased during pregnancy to about 0.5% of all reads at day 140 (Table 2). The close similarity of the coding sequences for *CSH2a,b,c, CSH4a,b,c*, and to a lesser extent *GH2a,b,c* made it impossible to distinguish between expression of these in many cases. *GH2* transcripts made up about 54% of the total *GH*-like transcripts, with *GH2a* being at least 60% of these. *CSH1* transcripts accounted for about 23% of the total and *CSH2* about 20%. Expression of *CSH4* was low (0-2% of all transcripts). There were no clear-cut differences between the proportions of transcripts at different developmental stages. No transcription of the putative pseudogene, *CSH3*, was detected, even when a sample of 300 000 placental transcripts was examined; expression is thus < 0.001% of all transcripts, lower than the 0.01% detected for the equivalent human pseudogene (2).

**Table 2.** Expression of *Macaca fascicularis* GH-like proteins in placenta

	Developmental stage					
	Days 20-35	Day 45	Day 62	Day 84	Day 90	Day 140
Total hits (reads)*^a^*	0	12 (12-12)	147 (126-168)	29 (27-30)	155 (135-174)	473 (345-600)
Total as % of all reads		0.012	0.147	0.029	0.155	0.473
Hits as % of total hits*^a^*						
CSH1	0	75 (50-100)	25 (20-29)	27 (20-33)	19 (11-26)	21 (18-23)
GH2a,b,c	0	25 (0-50)	56 (48-64)	69 (67-70)	46 (40-51)	50 (47-52)
CSH2a,b,c	0		15 (13-17)	0	19 (18-19)	27 (23-30)
CSH4a,b,c	0		1 (0-2)	0	2 (0-3)	0
Unassigned	0		4 (2-7)	5 (0-10)	12 (3-20)	4 (1-7)

Based on Sequence Read Archive project SRP329459 (31).

^*a*^Values are means (range) from 2 experiments.

### The *GH* Gene Cluster in Other *Macaca* Species

**Figure 3.**
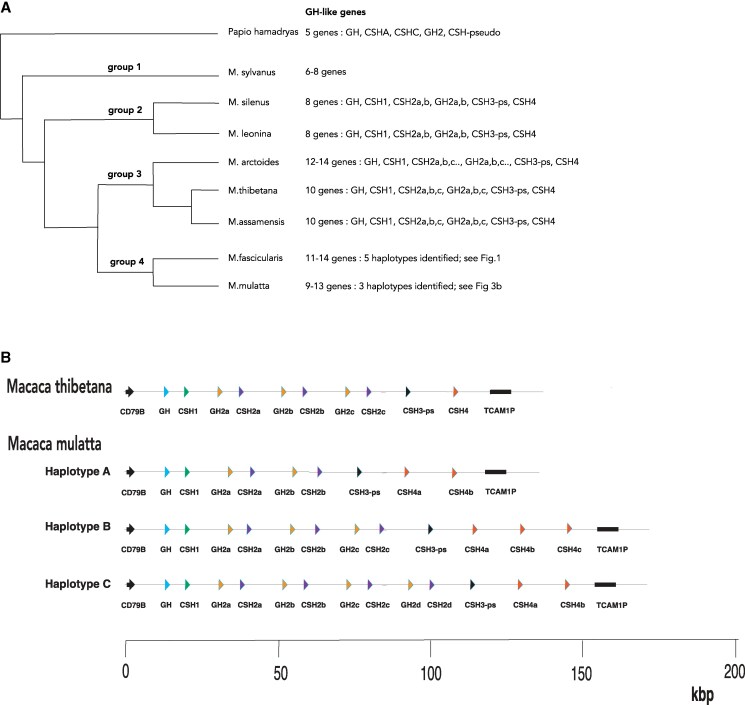
*GH*-like genes in various macaque species. (A) Phylogenetic tree summarizing evolutionary relationships within the genus *Macaca*. Tree based on Li et al (23). The number and nature of *GH*-like genes in each species are shown on the right-hand side. (B) *GH* gene cluster in *Macaca thibetana* and *Macaca mulatta*. *GH*-like genes are represented as triangles, distinguished by color and labeling. The gene for pituitary GH is at the 5′ end of the cluster (left-hand side) in every case. Flanking genes are also included: *CD79B* (5′) and *TCAM1P* (3′). The *Macaca thibetana* cluster is based on the reference sequence in the NCBI genome database. The *Macaca mulatta* haplotypes are based on an analysis of data in Sequence Read Archive project SRP477699.

The genus *Macaca* comprises at least 19 distinct species. These have been divided into 4 groups in a phylogenetic study based on *Alu* elements (23) (Fig. 3A). For 2 of these species, *M. fascicularis* (see previous discussion) and *M. mulatta*, sequence reads sufficiently long to include the entire *GH* gene cluster are available. For other species, representatives of the 4 groups, the number of genes in the cluster was estimated by Blast analysis of available short-read sequence databases and determining the numbers and proportions of hits on each sequence type (Table 3). Results are summarized in Fig. 3 and in the following for each group.

**Table 3.** Numbers and types of *GH*-like genes in various *Macaca* species

	*Macaca silenus*	*Macaca leonina*	*Macaca thibetana*	*Macaca assamensis*	*Macaca arctoides*	*Macaca fascicularis*	*Macaca mulatta*
GH	1.02 ± 0.10	1.00 ± 0.01	1.24	0.91 ± 0.07	0.99 ± 0.13	0.85 ± 0.08	1.01 ± 0.10
CSH1	0.97 ± 0.06	1.07 ± 0.04	1.04	0.96 ± 0.04	1.00 ± 0.14	0.92 ± 0.05	1.06 ± 0.07
GH2	2.28 ± 0.18	1.99 ± 0.12	3.57	3.06 ± 0.04	4.82 ± 0.62	2.71 ± 0.19	2.92 ± 0.21
CSH2	2.08 ± 0.13	2.04 ± 0.19	3.87	2.88 ± 0.13	4.56 ± 0.72	2.90 ± 0.31	2.78 ± 0.26
CSH3	1.01 ± 0.08	0.94 ± 0.05	0.72	1.13 ± 0.11	1.01 ± 0.05	1.23 ± 0.11	0.92 ± 0.06
CSH4	0.91 ± 0.14	0.92 ± 0.08	0.80	1.10 ± 0.12	1.15 ± 0.32	2.97 ± 0.36	2.98 ± 0.17
n	4	4	1	4	4	4	4
SRA Experiments	ERX10900094-7	ERX10899962-5	SRX8515195	ERX10900238-41	ERX10899922-5	ERX10899938-41	ERX10382739-42

SRA short-read experiments were analyzed by BLAST, using *Macaca fascicularis* GH exon 5 as Query. Hits on each GH-like gene type were recorded and normalized to GH:CSH1:CSH3 1:1:1. Values shown are means ± SEM for 4 replicates, except for *Macaca thibetana* for which only 1 experiment was analyzed.

Abbreviation: SRA, Sequence Read Archive.

#### Group 1

The only species included in this group is *Macaca sylvanus (M. sylvanus*), the barbary ape. No genome assembly has been reported for this species. Two short-read experiments in the SRA database provide sequence data but give conflicting results, 1 suggesting 6 *GH*-like genes, with just 1 copy of *GH2* and *CSH2*, and the other 8 *GH*-like genes, with 2 copies of *GH2* and *CSH2*. A clear conclusion cannot be reached about the situation in this species, and it is therefore not included further in the study.

#### Group 2

Two representatives were examined: *Macaca silenus* (*M. silenus*) and *Macaca leonina* (*M. leonina*). Gene assemblies available for these 2 species did not provide a useful basis for the current study (a very incomplete genome assembly is present in the NCBI database for *M. silenus*; no genome assembly is available for *M. leonina*). For each of these species, Blast analysis of short-read experiments indicated a *GH* gene cluster comprising 8 genes, with 2 copies of *GH2* and *CSH2* (Table 3, Fig. 3).

#### Group 3

Three representatives were examined: *Macaca thibetana* (*M. thibetana*)*, Macaca assamensis* (*M. assamensis*), and *Macaca arctoides* (*M. arctoides*). For *M. thibetana,* a genome assembly is present in the NCBI database; Blast analysis of this showed a cluster of 10 *GH*-like genes (including pituitary *GH*), organized as illustrated in Fig. 3B; this is equivalent to the 11-gene haplotype of *M. fascicularis*, without the duplication of the 3′-most gene (*CSH4*). Analysis of long sequence reads (SRA project SRP388525) (32) supports this genome assembly, but no read included the entire gene cluster, so the evidence is less strong than for *M. fascicularis*. Blast analysis of short-read experiments for this species indicated a *GH* gene cluster comprising 10 to 12 genes, with 3-4 copies of *GH2* and *CSH2* and only 1 of *CSH4* (Table 3, Fig. 3). An incomplete NCBI reference genome for *M. assamensis* is available; this contains only 2 *GH*-like sequences and therefore did not provide a useful basis for the current work. Blast analysis of short-read databases indicated 10 *GH*-like genes (Table 3, Fig. 3). The available NCBI genomic assembly for *M. arctoides* identified only 5 *GH*-related genes on several different contigs. Blast analysis of short-read experiments suggested 12 to 14 *GH*-like genes, similar to *M. thibetana* but with additional copies of *GH2* and *CSH2* (Table 3, Fig. 3).

#### Group 4

The 2 representatives for this group were *M. fascicularis* and *M. mulatta*. The organization of the *GH* gene cluster of the former has been considered already; Blast analysis of short-read experiments indicated a *GH* gene cluster with 12 genes for the individual examined (Table 3).

The *GH*-like genes in *M. mulatta* have been subject to previous study, and a number of conflicting genome assemblies have been reported. The NCBI reference genome (Mmul_10; also included in the Ensembl database) contains a cluster of 13 *GH*-like genes, with similar organization to that of *M. fascicularis* haplotype III; 2 of these genes showed multiple indels in the coding sequence, suggesting sequencing errors and indicating that this assembly did not provide a satisfactory basis for the current study. Analysis of original sequence data was therefore carried out. SRA Project SRP477699 includes long reads (Oxford Nanopore) for 5 *M. mulatta* individuals. A number of these reads include the full *GH* gene cluster for this species, which allowed identification of 3 different haplotypes for the locus, comprising 9, 12, or 13 *GH*-like genes, as illustrated in Fig. 3B. The most recent NCBI assembly (MacMul_CN_1; Jan. 2024) contains a cluster of 9 *GH*-like genes and appears to correspond to haplotype A (Fig. 3B).

These results indicate that there is considerable variation of the *GH* gene cluster within the genus *Macaca*. No evidence for more than 1 haplotype was found in species other than *M. fascicularis* and *M. mulatta*, but the number of individuals examined was small. Analysis of long-read sequences from these other species will be required to establish conclusively whether multiple haplotypes are only found in *M. fascicularis* and *M. mulatta*.

### Evolution of the *GH* Gene Cluster in Macaques

The evolution of the complex *GH* gene cluster in higher primates has involved repeated gene duplications, followed by divergent evolution. The resultant proteins show marked differences in terms of biological properties and expression patterns. As reported here, the expansion of the cluster in macaques (*Macaca*) is greater than that in other OWM/apes and has continued within this genus. Whether this expansion has been associated with changes in function is not clear.

Occurrence of adaptive evolution can be detected by analyzing coding sequences, in particular the ratio between changes in nonsynonymous (NonSyn) substitutions (which give rise to changed amino acid sequence) to synonymous (Syn) substitutions (which do not change this) (26). For most coding sequences, protein sequence is strongly conserved, and the NonSyn/Syn (dN/dS) ratio is low (<<1.0). If function is lost, the ratio approaches 1.0. If the gene is subject to adaptive evolution, the rate of NonSyn substitutions will increase. If the NonSyn/Syn ratio significantly exceeds 1.0, this is clear evidence for positive (adaptive) evolution, though failure to reach this exacting criterion does not prove the absence of adaptive evolution (25, 26).

Previous studies have shown the occurrence of adaptive evolution in the *GH* gene cluster in apes and OWM (3, 4, 19) on the basis of the NonSyn/Syn ratio > 1.0. Whether the extensive duplication and divergent evolution of the cluster among macaques also reflects adaptive evolution can similarly be tested in this way.

**Figure 4.**
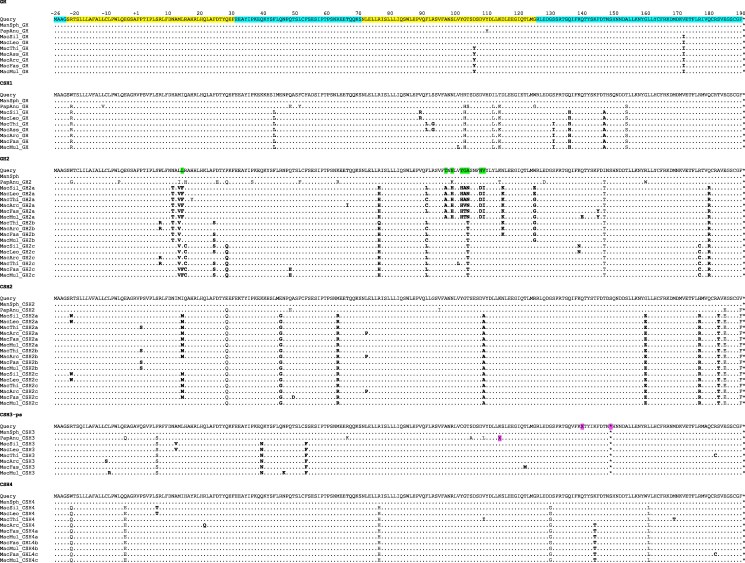
Alignments of GH-like protein sequences from *Macaca* species. Sequences of baboon (PapAnu) and mandrill (ManSph) proteins are used as outgroups, with the latter as Query. Cyan and yellow highlighting of GH indicates the 5 exons. The signal peptide is numbered −26 to −1. Residues highlighted in green indicate sites that change during the rapid evolution at the base of the GH2a clade. *CSH3* is probably a pseudogene, unlikely to be translated; its sequences have been “forced into frame” by introducing single nucleotide insertions at “X” and ignoring stop codons (*) (highlighted purple). Residues that changed at the base of or within the *Macaca* lineage are shown in bold. Abbreviations: MacArc, *Macaca arctoides*; MacAss, *Macaca assamensis*; MacFas, *Macaca fascicularis*; MacLeo, *Macaca leonina*; MacMul, *Macaca mulatta*; MacSil, *Macaca silenus*; MacThi, *Macaca thibetana*; ManSph, *Mandrillus sphinx*; PapHam, *Papio hamadryas*.

Figure 4 gives alignments of protein sequences derived from *GH*-like genes for 7 *Macaca* species that were discussed earlier. The corresponding sequences from mandrill (*Mandrillus sphinx*) and baboon (*Papio hamadryas*) are shown as outgroups, though for *CSH4*, there is no equivalent in baboon. Sequence variation within the *Macaca* genus is most marked for *GH2*, as has been noted previously for *M. fascicularis* (Fig. 2). *CSH2* and *CSH4* sequences are more strongly conserved.

**Figure 5.**
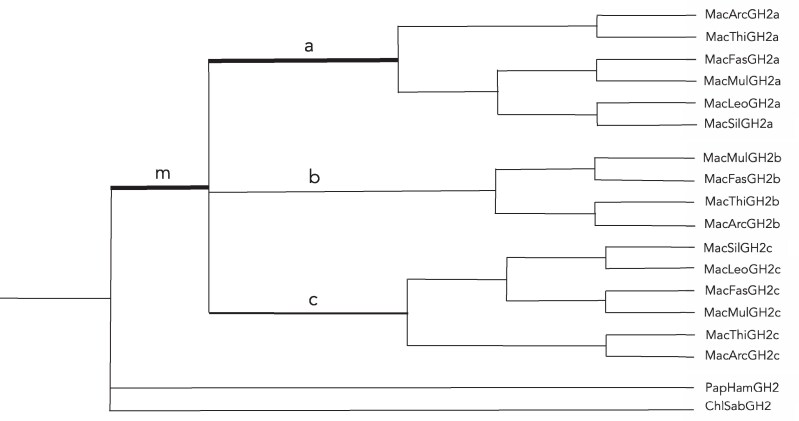
Phylogenetic tree illustrating the relationships between the various *Macaca GH2*-like genes. The thick branch at the base of the *GH2a* clade underwent an episode of rapid adaptive change, with very high dN/dS (10.1 NonSyn and 0 Syn substitutions; 2xΔlnL = 4.86, *P* < 0.05). Changes on branches a, c, and m are discussed in the text. Abbreviations: ChlSab, *Chlorocebus sabaeus*; MacArc, *Macaca arctoides*; MacFas, *Macaca fascicularis*; MacLeo, *Macaca leonina*; MacMul, *Macaca mulatta*; MacSil, *Macaca silenus*; MacThi, *Macaca thibetana*; PapHam, *Papio hamadryas*.

An alignment (Supplementary Fig. S1) (21) of *GH2* coding sequences was analyzed using the CODEML method (25), to assess whether adaptive evolution could be detected. A phylogenetic tree based on these sequences was obtained using PAUP (22); this grouped all *GH2a* and all *GH2c* sequences together, but the position of *GH2b* sequences was not clear, reflecting some (incomplete) similarity to both *GH2a* and *GH2c*. The branching order of *GH2a*, *GH2b,* and *GH2c* was therefore left undefined, as a polytomy (Fig. 5). CODEML analysis of the *GH2* sequences using this tree showed rapid evolution throughout the *Macaca* clade (dN/dS = 1.49) but not significantly greater than 1.0, the neutral rate. Evolution was particularly rapid on the basal branch for the *GH2a* clade (branch a in Fig. 5), with dN/dS significantly greater than 1 (dN/dS = 291; 10.1 NonSyn and 0 Syn substitutions; 2xΔlnL = 4.86, *P* < .05). There were relatively few changes on branches leading to *GH2b* and *GH2c* (branches b and c in Fig. 5), but 2 changes on branch c (R16C and R178C, numbering as in Fig. 4) potentially introduce a new disulfide bridge, unlikely to be a neutral change.

#### Location of residues changing with the appearance of GH2a

**Figure 6.**
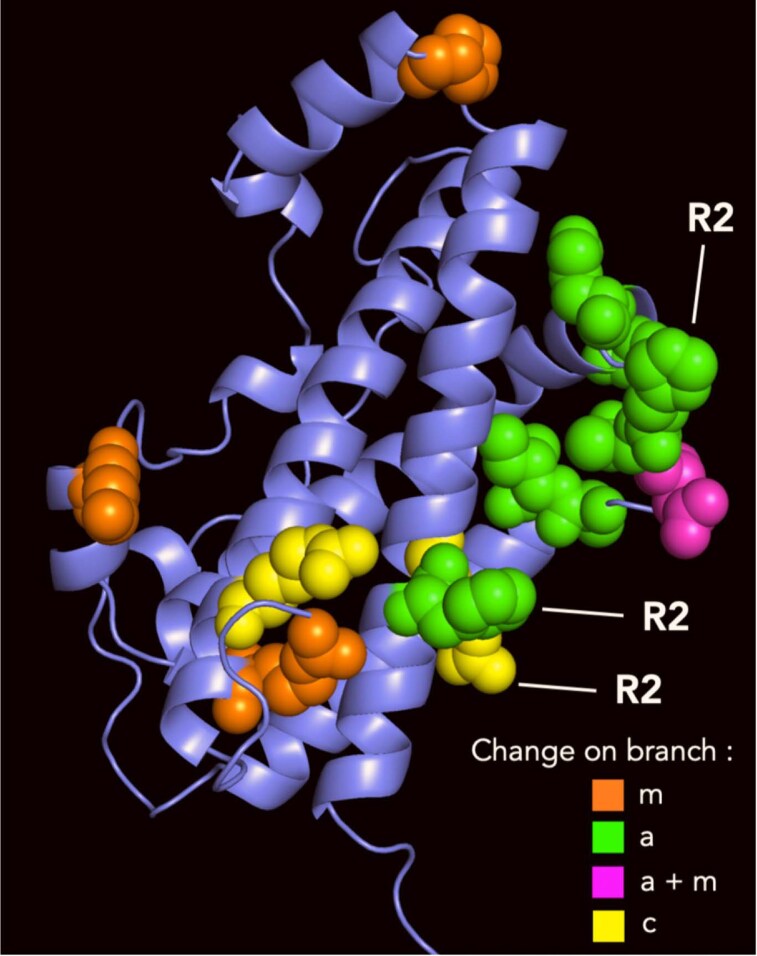
Molecular model of *Macaca fascicularis* GH2a. The protein backbone, with its characteristic 4-helix bundle, is slate blue. Residues shown in spacefill are those that change on specific branches of the phylogenetic tree (Fig. 5): branch a (green), m (orange), c (yellow), both a and m (magenta). Residues located in receptor-binding site 2, based on the human GH-receptor structure (28), are labeled R2.

The CODEML analysis identified an episode of rapid evolution that occurred after the gene duplication giving rise to *GH2a* but before subsequent diversification (Fig. 5, branch a). Eight unequivocal amino acid residue changes during this episode were identified using the APOLIST option in PAUP. A predicted 3D structure for *M. fascicularis* GH2a protein was constructed using AlphaFold 3, and the 8 changes were mapped onto this using PyMol (Fig. 6). Seven of these 8 residues are clustered closely in the short loop between helices 2 and 3 and the start of helix 3; 2 of them correspond to residues that are in the site 2 binding site in the human GH-receptor structure (28). The residues that change to cysteine on branch c in Fig. 5 are distant in the amino acid sequence but quite close in the 3D structure (Fig. 6), though modeling of GH2c using AlphaFold 3 suggested that they are not close enough to form a disulfide bond. Four residues show unambiguous changes on the branch leading to the *Macaca* clade (Fig. 5, branch m). These are scattered quite widely in the 3D structure; 1 also changes again on branch a (Fig. 6).

#### The GH receptor in macaques

The GH receptor was examined to determine whether observed changes in GH-like proteins were reflected in changes in the receptor. Blast searches of *Macaca* genomes using the human GH receptor CDS as probe detected, in every case, a single receptor gene, with no evidence for gene duplication. An alignment of macaque GH receptor amino acid sequences (all of the species shown in Fig. 3A; derived by conceptual translation of corresponding CDS) showed variation (substitutions) at 8 sites in the extracellular domain. These were distributed fairly evenly across the phylogenetic tree, with no suggestion of an episode of rapid evolution as seen for the GH-like proteins. Two of these 8 sites were close to bound GH in site 1 of the structural model of de Vos et al (28), but neither was identified as playing a substantial role in receptor binding in that study (28). The GH receptor sequence alignment is shown in Supplementary Fig. S2 (21); information about the data used to construct the alignment is given in the legend.

## Discussion

Expansion of gene number by gene duplication and subsequent divergent evolution has led to a *GH* locus that is more complex in higher primates than in other mammals, with the appearance of several genes expressed primarily in the placenta (1, 2). The initial gene duplication and subsequent expansion appears to have occurred independently in NWM and OWM/apes (5, 7, 8). In the latter group, the greatest expansion has occurred in macaques, leading to a cluster of 8 to 14 *GH*-like genes. The macaques comprise a large, widespread genus (*Macaca*), including models relevant to human physiology, and it is therefore important to clarify the nature of the *GH* cluster in these animals.

### The *GH* Gene Cluster in *M. fascicularis*

Detailed characterization of gene clusters in which the adjacent genes have very similar sequences, due to recent duplications and/or gene conversion, is notoriously difficult. That this applies in the case of the macaque *GH* gene locus is suggested by the disagreeing and incomplete gene assemblies that have been reported for this region and the observation that these assemblies include an abnormally high proportion of apparent pseudogenes, often associated with very frequent small indels, which are likely to reflect sequencing errors. In the case of *M. fascicularis* and *M. mulatta,* the availability in the SRA database of a number of experiments with very long (Oxford Nanopore) sequence reads (more than 150 kbp, sufficient to include the entire *GH* locus and flanking sequences) provided the basis for defining the overall organization of the *GH* locus in a number of individual animals. Not surprisingly, these long reads were relatively inaccurate, with many small indels, but the availability of shorter (but still more than 20 kbp), more accurate PacBio reads from the same individual animals allowed construction of an accurate sequence for 1 haplotype of the *M. fascicularis GH* gene cluster.

For *M. fascicularis,* analysis of the long sequence reads showed considerable heterozygosity. In particular, reads were identified containing 5 different haplotypes, I to V, with respectively 11, 12, 13, 14, and 13 *GH*-like genes, with the overall organization shown in Fig. 1. Individual animals contained either 1 or 2 haplotypes (Table 1). An individual (cy0325) that seemed to be homozygous for the 12-gene haplotype II was used to assemble the full sequence of the *GH* gene cluster (deposited as GenBank Accession number BK069932) using the more accurate PacBio sequence reads; these gave results consistent with a single haplotype in this individual. Striking features of the sequence were the almost exact repeats of long sequence (up to 20 kb) for *GH2a-CSH2a*, *GH2b-CSH2b,* and *GH2c-CSH2c* and for *CSH4a*, *CSH4b,* and *CSH4c*. Also notable is the large number of *Alu* sequences in the *GH* gene cluster, including a very recent insertion into intron 1 of *CSH4b*. This is the only *Alu* sequence (of a total of about 100 in the gene cluster) to occur within an intron rather than in intergene regions; it more than doubles the length of intron 1 in *CSH4b* and is only found in haplotype II. In other respects, the density and distribution of *Alu* elements is similar to that in ape (including human) *GH* gene clusters (2, 16) and comprises about 20% of the total sequence.

Alignment of protein sequences obtained by conceptual translation of the *M. fascicularis GH*-like genes showed that they correspond to several previously recognized groups. The gene for pituitary GH is at the 5′ end of the cluster, as is the case in human and all other characterized higher primate *GH* loci. The encoded protein sequence is very similar to that of human GH. Proteins encoded by genes *CSH1*, *CSH2a*, *CSH2b*, *CSH2c*, *CSH4a*, *CSH4b*, and *CSH4c* have similar sequences, resembling that of human *CSH1* and *CSH2*, and presumably express the macaque equivalents of human placental lactogen. These 7 macaque genes fall into 3 groups, *CSH1*, *CSH2a,b,c,* and *CSH4a,b,c*; the sequences of *CSH2a,b* and *c* and especially *CSH4a,b* and *c* are very similar to each other. *GH2a*, *GH2b,* and *GH2c* encode similar proteins, equivalent to the *GH2* reported previously in other OWM. Sequences corresponding to *CSH1*, *GH2*, *CSH2,* and *CSH4* have been described previously from *M. mulatta* (18), but the occurrence of multiple forms of the last 3 genes has not. *CSH3* of *M. fascicularis* is apparently a pseudogene.

### The *GH* Gene Cluster in Other *Macaca* species

The availability of sequence reads long enough to include the entire *GH* gene cluster in *M. fascicularis* enabled the complexities of the *GH* locus in this species to be resolved, at least in part. Such long reads are also available for *M. mulatta* and enabled 3 different haplotypes for the *GH* gene cluster in this species to be identified (Fig. 3B). The error rate in these long-read experiments was high, and all of the individuals examined appeared to be heterozygotes, with 2 different haplotypes, making it difficult to establish an accurate complete sequence for any 1 haplotype.

For other *Macaca* species, long reads including the entire gene cluster were not available. However, short-read experiments are available for a number of species, and from these the numbers and types of *GH* gene sequences and genes could be determined (Table 3, Fig. 3A), as well as, in many cases, detailed coding sequences for individual genes. This gave information for representatives of each of the 4 main species groups of *Macaca* (23, 24) (Fig 3A).

Group 1 includes a single species, *M. sylvanus*, for which 2 genomic sequencing experiments are available. These gave differing results for the number of *GH-*like genes: 6 in 1 case and 8 in the other. Two species were analyzed in group 2: *M. leonina* and *M. silenus.* Each of these had 8 *GH*-like genes, including duplicates of *GH*2 and *CSH2* and a single copy of *CSH4*. The species studied from group 3 had 10 (*M. thibetana* and *M. assamensis*) or 12 to 14 (*M. arctoides*) *GH*-like genes, reflecting further duplications of *GH2* and *CSH2,* but only a single *CSH4*. Group 4, including *M. fasciculari*s and *M. mulatta*, had 9 to 14 *GH*-like genes, reflecting the multiple haplotypes detected here. Only in this group are there multiple copies of *CSH4*. Thus, the complexity of the *GH* gene cluster seems to increase with evolution of the *Macaca* genus. Whether the multiple haplotypes seen in group 4 also occur in other species cannot be determined from the available data, but it could underlie some of the variability seen in some cases, such as *M. arctoides*. Full characterization of the *GH* gene cluster in *Macaca* species other than *M. fascicularis* and *M. mulatta* will require the availability of long-read sequences including the entire cluster.

### Biological Role and Evolution of *GH*-like Genes in *Macaca*

In human, the *GH* locus contains 5 *GH*-like genes, but these give rise to only 3 GH-like proteins: pituitary GH and 2 proteins expressed in the placenta, PL/CSH, expressed from 2 almost identical genes, and GH-variant/GH2. The fifth gene is probably a pseudogene. GH2 is expressed at a low level, achieves a moderate concentration in the maternal circulation, and appears to replace pituitary GH during pregnancy. Human CSH is expressed at a high level and reaches a very high circulating concentration during pregnancy (33); its physiological role is not clear.

In OWM the *GH* locus is at least as complex as in human, comprising genes encoding pituitary GH, at least 2 distinct CSHs, and GH2 (6, 19). The GH2 of OWM is substantially different from human GH2; they may not be strictly orthologous. It is shown in this paper that the complexity of the *GH* locus has increased further in macaques, with the number of *GH*-like genes increasing from 6 to 8 in the basal group (*M. sylvanus*) to at least 14 in some species, such as *M. fascicularis*. This has involved additional duplication(s) of *GH2-CSH2* (as a pair) and *CSH4*, resulting in up to 4 or 5 copies of *GH2-CSH2* and 3 of *CSH4*. That this rapid evolution of the locus is still continuing in some macaques is suggested by the observation that the locus is heterozygous in *M. fascicularis* and *M. mulatta*, with multiple haplotypes containing 11 to 14 genes in the former and 9 to 13 in the latter.

It is clear that the *GH*-locus has expanded within the *Macaca* genus and that the evolutionary trend is continuing within at least some *Macaca* species. At least 2 possible explanations can be considered. First, it may reflect the natural tendency for duplicate genes to undergo further duplications because of increased mismatch recombination between the duplicated sequences (34). The additional genes would have no adaptive significance and reflect neutral evolution. The expansion would eventually be limited because the same mismatch recombination mechanism can lead to gene deletion as well as duplication; this could be the explanation for the occurrence of a haplotype in *M. mulatta* with only 9 *GH*-like genes, when all other species in groups 3 and 4 (Fig. 3A) have at least 10.

An alternative explanation for the expansion of the *GH* locus is that it results from adaptive evolution, with duplicate genes, arising by mismatch recombination, being retained because they have selective advantage. The CODEML application in the PAML package (25, 26) provides a method for testing for positive selection and has been used previously to detect such selection in the evolution of *GH*-like genes in OWM/apes (19). In the present study, it was used to show positive selection during the episode of rapid evolution of the *GH2a* gene in macaques (Fig. 5). The observation that most of the changes that were introduced into the protein during this episode were located in a fairly small region of the 3D structure, close to receptor-binding site 2 (Fig. 6), also suggests adaptive evolution, though examination of the receptor did not suggest coevolution between ligands and receptor. Rather few substitutions were introduced into the sequence of GH2c after divergence from GH2a, but 2 of these (R16C and R178C, Fig. 4) involve introduction of cysteine residues into receptor-binding site 1 (R178C) and binding site 2 (R16C) and are therefore likely to affect biological function. CSH2 and CSH4 show few changes following duplication, and here the case for adaptive evolution is less clear. Why GH2 shows more change than CSH2 or CSH4 is not clear. Duplication of the *CSH2* and *GH2* genes usually occurs together, as a pair, and this may be driven by selection operating on the *GH2* genes, with the *CSH2* genes following on. Duplication of *CSH4* genes occurs independently of *GH2*, however. Expression levels of *CSH4* are low, and it is possible that duplication of the gene was driven by selection for greater expression. The driving force for the adaptive evolution of the *GH2* genes in macaques, as indicated by the CODEML analysis, is unclear, particularly given that the physiological role of the GH2 protein in OWM is poorly understood. The biological significance is discussed further below.

The biological role and function of the placental GH-like proteins in higher primates remains poorly defined (10). A role in promoting mammary growth and development during pregnancy is possible but has to be reconciled with their abrupt disappearance at parturition, the time when the main function of the mammary gland begins. A role in regulating the balance of nutrition between mother and fetus(es) could also be important, in which context the fact that GH-like proteins are produced in the fetal side of the placenta may be significant, raising the possibility of evolution of the proteins being driven by fetal-maternal competition (11). The possibility of a role for the proteins in protecting the fetus against pathogens has also been raised (13). In considering these biological roles, 2 remarkable features have to be considered. First is the extremely high concentrations of at least some of the GH-like proteins that occur in the maternal circulation (∼100 fold greater than normal circulating concentration of GH and prolactin), and second is the complexity and diversity of the *GH* gene cluster, seen at its extreme in the expansions seen in macaques and some NWM. In the case of the macaques, expansion of the gene cluster has been very recent, with 5 to 7 genes in the most recent OWM relatives of this genus and 8 to 14 in macaques. Such rapid evolution can often reflect a host-pathogen arms race (13). Some hormone receptors are used by viruses to facilitate cell entry, and although this has not been shown for GH, prolactin, or their receptors (35), it is notable that the protein nectin-4, which interacts with the prolactin receptor (36, 37), is used in this way by some viruses, including measles (38). Why the *GH* gene cluster should have become so diverse and complex in macaques is unclear. *Macaca* is a very successful genus, containing many distinct species, with a broad geographic distribution and living in a diverse range of environmental conditions. Which of these factors may have contributed to the variation and complexity of the *GH-*like genes is for the present a matter for speculation.

Although they have been known for over 50 years, the role of the GH-related placental proteins in human, particularly PL, remains an important unanswered question in human physiology. Given that equivalent proteins only occur in higher primates, elucidation of the nature of the *GH* gene cluster in macaques may provide a step toward solving the problem. The size and complexity of the cluster are greater in macaques, but the most notable features of the cluster—placental expression at very high levels and rapid evolutionary change—are common to both monkeys and human.

## Disclosures

The author has nothing to disclose.

## Data Availability

Original data generated and analyzed during this study are included in this published article or in the data repositories listed in the references.
